# Circulating miR-1 as a potential biomarker of doxorubicin-induced cardiotoxicity in breast cancer patients

**DOI:** 10.18632/oncotarget.14355

**Published:** 2016-12-29

**Authors:** Rigaud Oliveira-Carvalho Vagner, Ludmila R.P Ferreira, Silvia M Ayub-Ferreira, Mônica S Ávila, Sara M.G Brandão, Fátima D Cruz, Marília H.H Santos, Cecilia B.B.V Cruz, Marco S.L Alves, Victor S Issa, Guilherme V Guimarães, Edécio Cunha-Neto, Edimar A Bocchi

**Affiliations:** ^1^ Heart Failure Unit, Heart Institute (InCor), Hospital das Clínicas da Faculdade de Medicina da Universidade de São Paulo (HC-FMUSP), São Paulo, Brazil; ^2^ Laboratory of Immunology, Heart Institute (InCor), Hospital das Clínicas da Faculdade de Medicina da Universidade de São Paulo (HC-FMUSP), São Paulo, Brazil; ^3^ University of Santo Amaro (UNISA), Hospital das Clínicas da Faculdade de Medicina da Universidade de São Paulo (HC-FMUSP), São Paulo, Brazil

**Keywords:** microRNAs, doxorubicin, breast neoplasms, cardiotoxicity, biomarkers

## Abstract

Cardiotoxicity is associated with the chronic use of doxorubicin leading to cardiomyopathy and heart failure. Identification of cardiotoxicity-specific miRNA biomarkers could provide clinicians with a valuable prognostic tool. The aim of the study was to evaluate circulating levels of miRNAs in breast cancer patients receiving doxorubicin treatment and to correlate with cardiac function. This is an ancillary study from “Carvedilol Effect on Chemotherapy-induced Cardiotoxicity” (CECCY trial), which included 56 female patients (49.9±3.3 years of age) from the placebo arm. Enrolled patients were treated with doxorubicin followed by taxanes. cTnI, LVEF, and miRNAs were measured periodically. Circulating levels of miR-1, -133b, -146a, and -423-5p increased during the treatment whereas miR-208a and -208b were undetectable. cTnI increased from 6.6±0.3 to 46.7±5.5 pg/mL (p<0.001), while overall LVEF tended to decrease from 65.3±0.5 to 63.8±0.9 (p=0.053) over 12 months. Ten patients (17.9%) developed cardiotoxicity showing a decrease in LVEF from 67.2±1.0 to 58.8±2.7 (p=0.005). miR-1 was associated with changes in LVEF (r=-0.531, p<0.001). In a ROC curve analysis miR-1 showed an AUC greater than cTnI to discriminate between patients who did and did not develop cardiotoxicity (AUC = 0.851 and 0.544, p= 0.0016). Our data suggest that circulating miR-1 might be a potential new biomarker of doxorubicin-induced cardiotoxicity in breast cancer patients.

## INTRODUCTION

Breast cancer is one of the most common cancers among women worldwide and is usually treated with the potent anticancer drug doxorubicin (DOX). Despite its beneficial effect, the pro-apoptotic effect of this chemotherapy is not cancer-cell specific, and, therefore, the harmful effect of the anticancer drugs also reaches nonmalignant cells, such as cardiomyocytes [1]. This toxicity may compromise the clinical effectiveness of chemotherapy, independently of the oncologic prognosis, and impact the patient's survival and quality of life. Indeed, the chronic use of DOX is associated with a high risk of developing cardiotoxicity that is progressive and can develop into cardiomyopathy and heart failure up to 20 years after the end of the treatment [2].

These unwanted side effects have been known for several decades; however, it is still difficult to assess the risk of long-term cardiotoxicity induced by DOX in a timely manner and to prevent and/or reduce cardiac damage. Currently, drug-induced cardiotoxicity is clinically assessed by regular evaluation of left ventricle ejection fraction (LVEF) by imaging techniques. However, the cardiac damage is usually detected late when a functional impairment has already occurred, not allowing for early preventive strategies. Thus, the development of clinically validated early subclinical myocardial injury biomarkers is a primary goal for both cardiologists and oncologists, allowing the monitoring of the progression of cardiac damage and the planning of therapeutic strategies to support cardiac function.

microRNAs (miRNAs) are small non-protein-coded RNAs involved in the regulation of gene expression. During the last decade, these molecules garnered much attention for their potential as promising new biomarkers for a wide range of diseases with the benefits of both tissue and disease-specific expression signatures and high stability in body fluids [3]. Recently, some circulating miRNAs such as miR-1, -146a, -133b, -208a, -208b and -423-5p have been suggested as earlier biomarkers of myocardial injury, heart failure and/or drug-induced cardiotoxicity [4, 5]. However, until now, the potential of these circulating miRNAs as biomarkers of DOX-induced cardiotoxicity in a clinical setting has not been assessed. Therefore, the aim of this study was to evaluate circulating levels of miR-1, -133b, -146a, -208a, -208b, and -423-5p in breast cancer patients receiving DOX treatment and to correlate its levels with cardiac function.

## RESULTS

### Study population

A total of 56 female patients (49.9±3.3 age) were enrolled in this study. The first patient in the placebo arm was enrolled in April 2013, and the last analyzed was in October 2015. They received a total dosage of 410.3±9.5mg DOX in 4 cycles for 3 months. The clinical characteristics of the population are summarized in the Table 1.

**Table 1:** Patient characteristics

	Entire cohort (n=56)	Cardiotoxicity (n=10)	No-cardiotoxicity (n=46)
Age, y	49.9±3.3	48.6±3.2	49.9±1.2
Hypertension	5 (8.5)	1 (10)	5 (10.9)
Diabetes mellitus	1 (1.7)	0 (0)	1 (2.2)
Current or past smokers	17 (28.8)	5 (50)	13 (28.3)
Body mass index, kg/m^2^	28.5±3.3	27.5±6.5	29.4±1.1
Doxorubicin total dose, mg/m^2^	410.3±9.5	408.4±1.4	410.6±11.4
Menopause			
Pre	29 (49.2)	5 (50)	22 (47.8)
Post	30 (50.8)	5 (50)	24 (52.2)
Breast cancer side			
Right	28 (47.5)	4 (40)	21 (45.7)
Left	30 (50.8)	6 (60)	24 (52.2)
Bilateral	1 (1.7)	0 (0)	1 (2.2)
Baseline LVEF, %	65.3±0.5	67.2±1.0	64.9±0.5

Values are expressed as mean±SEM or n (%); LVEF - Left ventricle ejection fraction.

### Effect of DOX treatment on cTnI and LVEF

**Figure 1:**
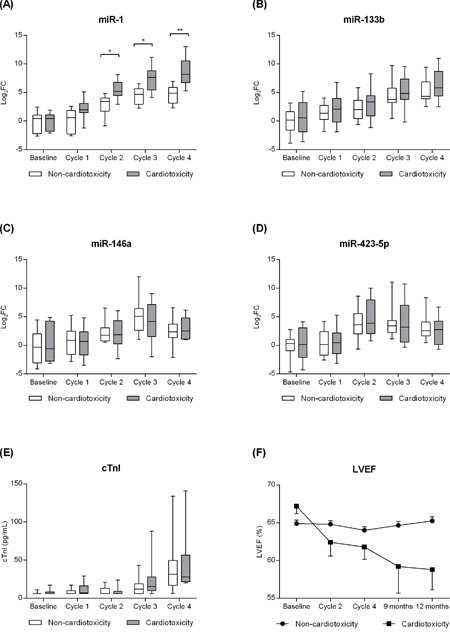
Effect of doxorubicin treatment on circulating levels of **A**. miR-1, **B**. -133b, **C**. -146a, and **D**. -423-5p; **E**. plasma levels of cardiac troponin I (cTnI); **F**. and left ventricle ejection fraction (LVEF) in patients with (n=10) and without cardiotoxicity (n=46). *p<0.05, **p<0.01.

**Figure 2:**
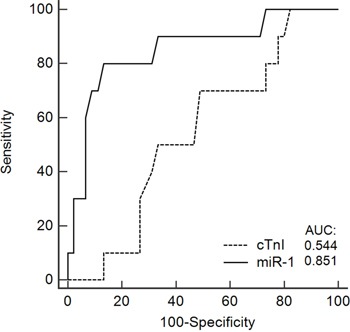
Associations between changes in Left Ventricle Ejection Fraction (ΔLVEF/LVEFbaseline) with the circulating level of **A**. miR-1; **B**. -133b; **C**. -146a; **D**. -423-5p; and **E**. cardiac troponin I (cTnI) in patients with breast cancer treated with doxorubicin.

Plasma levels of cTnI increased in each cycle from the baseline (6.64±0.3 pg/mL) until the end of DOX treatment (46.0±5.5 pg/mL; Figure 1). On the other hand, LVEF decreased over 12 months by an average of 2% when the group was analyzed as a whole, however, no statistical significance was observed. Baseline LVEF decreased from 65.3±0.5 to 64.3±0.6 at cycle 4 of the DOX treatment (p=0.549) and to 63.8±0.9 (p=0.053) after 12 months (Figure 1) with a tendency toward significance. However, an association was not observed between changes in LVEF and cTnI levels (Figure 2).

A total of 10 (17.9%) patients met the criteria for cardiotoxicity (reduction in LVEF ≥10% and/or LVEF <50%) with a mean of 7.2±1.3 months after chemotherapy initiation. Seventy percent (7/10) of the patients met the criteria for cardiotoxicity in a late stage - only after the end of the DOX treatment, which is consistent to previous studies. Within the cardiotoxicity group, LVEF decreased from 67.2±1.0 (baseline) to 58.8±2.7 (12 months; p=0.005) while remained preserved in non-cardiotoxicity patients (from 64.9±0.5 to 65.2±0.6; p=0.962). A difference in plasma levels of cTnI between cardiotoxicity and non-cardiotoxicity patients was not observed during the entire DOX treatment (Figure 1).

### Effect of DOX treatment on the circulating levels of miR-1, -133b, -146a, -208a/b and -423-5p

An overall increase of the circulating levels of miR-1, -133b, -146a, and -423-5p was observed during the DOX treatment (Figure 1). On the other hand, miR-208a was undetected during the entire treatment, and miR-208b had C_t_ values above 38 also being considered undetected. Comparing patients who did and did not developed cardiotoxicity, only miR-1 showed a differential pattern after cycle 2 being significantly more expressed in cardiotoxicity patients (Figure 1A). On the other hand, a significant difference in the circulating levels of miR-133b, -146a, and -423-5p between cardiotoxicity and non-cardiotoxicity patients was not observed during the entire DOX treatment.

**Figure 3:**
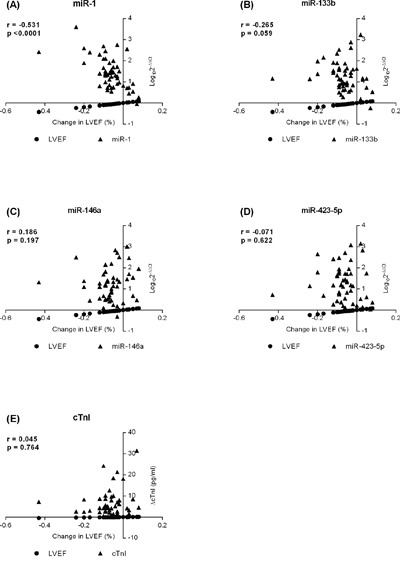
A receiver operating characteristic (ROC) curve predicting the ability of the circulating levels of miR-1 and cardiac troponin I (cTnI) to identify patients with cardiotoxicity from those with no cardiotoxicity Full line represents the area under the curve (AUC) of 0.851 (95% confidence interval, 0.729–0.933) for miR-1 and the dashed line an AUC of 0.544 (95% CI, 0.405-0.679) for cTnI. A pairwise comparison of ROC curves showed a difference between areas of 0.307 (95% CI, 0.116-0.497) and a p-value of 0.0016.

In a Spearman's rank coefficient analysis, the circulating level of miR-1 was associated with changes in LVEF (r =-0.531, p<0.0001), whereas no association was observed for miR-133b, -146a, and -423-5p (Figure 2). ROC curves were generated to predict the potential of the circulating levels of miR-1 and cTnI to detect cardiotoxicity, revealing an area under the curve of 0.851 (95% confidence interval, 0.729–0.933) and 0.544 (95% CI, 0.405-0.679), respectively. A pairwise comparison of ROC curves showed a difference between areas of 0.307 (95% CI, 0.116-0.497) and a p-value of 0.0016 (Figure 3).

## DISCUSSION

To our knowledge, this investigation is the first to demonstrate that circulating miR-1 is associated with changes in LVEF in patients receiving DOX treatment. ROC curve analysis showed that miR-1 was superior to cTnI for distinguishing between patients who did and did not develop cardiotoxicity, suggesting that miR-1 may become a potential biomarker of cardiotoxicity in patients undergoing DOX treatment. Also, we demonstrated that circulating levels of -133b, -146a, and -423-5p were upregulated in response to DOX treatment in patients with breast cancer.

### MicroRNAs: miR-1, -133b, -146a, -208a/b and -423-5p

The data presented in this study confirm previous reports showing that DOX treatment can perturb the expression of cardiac-related miRNAs [4, 5, 9]. Indeed, very few studies have evaluated miRNA expression patterns during DOX-induced cardiotoxicity, concentrating essentially on experimental approaches in mice or cell culture. Supporting our study, Horie et al [10] showed that miR-146a was significantly upregulated in rat cardiomyocyte cultures after a single high DOX dose inducing cell death via inhibition of ErbB4 gene. This is an interesting finding, because the inhibition of the ErbB4 gene by DOX was postulated to be involved in the development of heart failure in DOX-treated cancer patients. On the other hand, in a chronic model of cardiac toxicity, Vacchi-Suzzi et al [9] showed that miR-146a was not altered in mice hearts even with the evidence of histopathological lesion. In a clinical setting, we showed that the circulating levels of miR-146a increased significantly after the second DOX administration. In fact, we do not know whether miR-146a was released by the heart and whether this increase might be related to an early molecular stage of cardiomyocyte damage.

In agreement with our findings, upregulation of the muscle-specific miR-1 and -133b in plasma was also reported after DOX administration in an animal model [5], while no significant changes were observed in mice hearts [4, 9]. Taken together, the maintaining of miR-1 levels in the heart and its progressive increase in plasma suggest an active release of miR-1 from the heart that is consistent with the increase of cardiomyocyte vacuolation reported after DOX treatment [4, 9].

To date, many studies have highlighted the potential of the circulating levels of miR-423-5p as a biomarker of heart failure, correlating with important clinical prognostic parameters [11–15]. However, a significant upregulation of miR-423-5p was observed in the present study without a significant association with LVEF. Because miR-423-5p overexpression was reported as sufficient to induce apoptosis in cardiomyocytes [16], its increased level in the plasma of DOX-treated patients might be an indication of an earlier molecular change in the heart. It therefore remains elusive whether this increased miR-423-5p level is caused by cardiomyocyte death or whether it is actively released by other cell types in response of DOX.

Interestingly, miR-208a (heart-specific miRNA) [17] and -208b were undetected in this study, although previously published studies have suggested them as new biomarkers of myocardial injury following myocardial infarction [18, 19] or isoproterenol use [5, 20, 21]. This discrepancy may be a result of a differential response of the cardiomyocytes to DOX stimuli. As shown by Vacchi-Suzzi et al [9], miR-208a decreases during DOX-treatment similarly to its encoding gene Myh6, while miR-208b and Myh7 (its encoding gene) increase their levels in parallel indicating a myosin switch that is associated with pathological cardiac remodeling [17]. Supporting our data, Nishimura et al [5] showed that after the single administration of a high DOX dose in rats, the circulating level of miR-208a did not change significantly, but miR-1, miR-133a/b, and miR-206 increased. Taken together, apparently DOX-induced myocardial injury occurs in a different pathway than that observed in myocardial infarction and isoproterenol-induced myocardial injury, which partially explains the lack of miR-208a in the plasma of DOX-treated patients.

### MicroRNA-1 and cardiotoxicity

The occurrence of DOX-induced cardiotoxicity is widely variable, depending on several risk factors, such as the cumulative DOX dose administrated, age, and concomitant treatment with drugs like trastuzumab [8]. In our study, we observed an occurrence of cardiotoxicity in 17.9% of the patients receiving cumulative doses considered as “safe” and an overall reduction of 2% in LVEF when analyzing the entire cohort over 12 months. This reduction in LVEF did not reach statistical significance, probably due to the small sample size, but it was consistent with the reduction of similar magnitude (2.6%) observed in the recent PRADA trial [22].

In our study, miR-1 showed a superior ability than cTnI to discriminate between patients with and without cardiotoxicity induced by DOX treatment. cTnI has been shown primarily by Cardinale et al. [23, 24] to be a biomarker of cardiotoxicity in patients receiving high-dose anthracyclines. However, most of those patients had hematologic malignancies and received higher doses than breast cancer patients in our study. This difference could help to explain the relative lack of predictive utility of cTnI found in our study and highlights the potential of miR-1 as a biomarker.

Despite many studies having explored the potential of miR-1 as a biomarker of myocardial injury [5, 25–28], to our knowledge, this is the first study to show a moderate association between miR-1 and changes in LVEF and its potential as a biomarker of earlier DOX-induced cardiotoxicity. This association might be partially explained by its recently reported involvement in reactive oxygen species (ROS) generation and subsequent oxidative stress-induced cardiomyocyte apoptosis - one of the main mechanisms of DOX-induced cardiotoxicity [1, 29, 30]. Indeed, previous studies [29–33] have shown that miR-1 suppresses the expression of antioxidant genes in cardiomyocytes that might lead to an imbalance between oxidative stress and antioxidant activities and a subsequent apoptosis that might elevate the circulating level of miR-1.

The miR-1 upregulation in DOX-treated patients coupled with its pro-apoptotic role via ROS generation opens a new window about its use as a therapeutic target to repress the cardiotoxic effect of the chemotherapy. Indeed, many miRNA-based therapies have been recently studied in cardiovascular diseases, resulting in promising findings to improve or protect heart functions [34–36]. Therefore, our results highlight not only the potential of miR-1 as promising new biomarker of DOX-induced myocardial injury, but also the possible use of miR-1 as a therapeutic target.

In this investigation we showed for the first time that the increase of circulating miR-1 is associated with cardiac dysfunction in patients with breast cancer undergoing sequential therapy with doxorubicin. Furthermore, miR-1 revealed a superior ability to cTnI for distinguishing between patients who did and did not develop cardiotoxicity raising its potential as a promising new biomarker. These results may lead to new earlier strategies to detect and monitor drug-induced cardiac damage before it progresses to an irreversible stage.

### Limitations

Regardless of the high accuracy of the individual RT-qPCR tests, their use restricted the data to only a few miRNAs not allowing a more comprehensive profile of miRNAs. Since miR-1 is produced by both cardiac and skeletal muscles, we do not know where miR-1 originates from. Another limitation is that high-sensitivity troponin levels were not determined in our study. Finally, a longer follow-up of these patients is still required to determine whether the increase in miRNAs is predictive of cardiac events in later years.

Additional studies are still required to validate the findings and provide mechanistic insight. Data presented are from a single cohort of patients and confirmatory findings from an additional, separate cohort of patients will significantly add to the validity of the results. Mechanistic validation by additional experiments is needed to determine the source and mechanism of the observed changes in miRNAs levels.

## MATERIALS AND METHODS

This is an ancillary and prospective study from the clinical trial “Carvedilol Effect on Chemotherapy-induced Cardiotoxicity” ( ClinicalTrials.gov NCT01724450; CECCY trial). Patient randomization was provided by the security committee preserving the study's double blindness. The trial, including enrollment of patients, is ongoing. This is an interim analysis of the placebo arm and only the reported variables were analyzed. The study protocol was approved by the ethics committee of the institution, and all subjects gave informed consent. No racial/ethnic-based differences were present.

### Inclusion criteria

Patients enrolled in this study had been diagnosed with breast adenocarcinoma, were indicated for doxorubicin treatment, had no history of cardiovascular disease, and were ≥18 years of age. Patients were excluded if they had a history of chemotherapy or radiotherapy, were HER-2 positive, β-blocker contraindicated, or used ACE inhibitors, angiotensin II receptor blockers, or β-blockers.

### Study design

Only the patients from the placebo group of the CECCY trial were included in this study. All enrolled patients underwent chemotherapy with 4 cycles of 60mg/m^2^ doxorubicin (cumulative dose of 240mg/m^2^) and 600mg/m^2^ cyclophosphamide every 3 weeks followed by paclitaxel 80 mg/m^2^ for 12 weeks or docetaxel 75 mg/m^2^ every 3 weeks. Blood samples were collected 3 weeks after each cycle for assessment of miR-1, miR-133b, miR-146a, miR-208a, miR-208b, miR-423-5p, and cardiac troponin I (cTnI). Cardiac function was assessed by doppler echocardiography (Simpson method) at baseline, twice during DOX treatment (cycle 2 and 4), and 9 and 12 months after the beginning of the chemotherapy. All measurements were performed in a blinded fashion following current guidelines [6, 7]. The doppler echocardiograms were analyzed independently and blindly by two cardiologists specialized in echocardiography. In face of a disagreement between the two examiners, a third cardiologist specialized in echocardiography was available for a blind review. Cardiotoxicity was defined as a reduction in LVEF ≥10% and/or LVEF <50% on any echocardiogram performed during or after the chemotherapy [8]. The plasma level of cTnI was assessed by automated chemiluminescence assay (Advia Centaur, Siemens).

### Blood sampling

Blood samples were collected by venous puncture into 4mL vacuum tubes containing EDTA. Plasma was obtained by centrifugation at 2000g at 4°C for 15 minutes, and the supernatant was carefully transferred to a new RNAse/DNAse-free 1.5mL microtube and stored at -80°C until use.

### RNA isolation and qPCR

Total RNA was isolated using the miRNeasy Serum/Plasma kit (Qiagen), according to the manufacturer's instructions. cDNA were synthesized by reverse transcription using a fixed volume of RNA (2μL) and the TaqMan microRNA Reverse Transcription kit (Life Technologies), according to the manufacturer's instructions. Circulating miRNAs were measured by RT-qPCR using 1.33μL of the cDNA and miRNA-specific stem-loop primers provided by TaqMan microRNA Assays kit (Life Technologies). Quantitative PCR reactions were performed in triplicate on a QuantiStudio 12K Flex (Life Technologies), according to the following program: 10 minutes at 95°C, 40 cycles of 15 seconds at 95°C and 60 seconds at 60°C. Values were normalized to cel-miR-39, analyzed by the comparative method of C_t_ (2^-ΔΔCt^) and expressed in log_10_.

### Statistical analysis

All results are expressed as means±SEM, if not stated otherwise. Data were statistically analyzed with GraphPad Prism 6 for Windows by using two-way ANOVA (repeated measures) with Sidak's multiple comparison test. Spearman's rank correlation coefficient was calculated to estimate the associations between circulating levels of miRNAs, cTnI and changes in LVEF (ΔLVEF/LVEF_baseline_). Receiver operator characteristic (ROC) analysis was generated to determine the ability of miRNA and cTnI levels to discriminate between cardiotoxicity and no-cardiotoxicity patients. The difference between the ROC curves was compared using DeLong test in MedCalc v.16.8.4. All tests were performed 2-tailed, and a significance level of p<0.05 was considered to indicate statistical significance. To verify whether studied miRNAs were differentially expressed in patients with cardiotoxicity, we grouped those patients who met the cardiotoxicity criteria (described above) as “cardiotoxicity group” and compared them with the others (“non-cardiotoxicity group”).
